# Effects of Tumor-derived Small Extracellular Vesicles on T cell Survival in Patients with Cancer; A Commentary

**DOI:** 10.33696/cancerimmunol.6.097

**Published:** 2024

**Authors:** Theresa L. Whiteside

**Affiliations:** 1 Department of Pathology, University of Pittsburgh School of Medicine, Pittsburgh, PA 15213, USA

**Keywords:** Small extracellular vesicles, Tumor derived exosomes (TEX), Cancer, T cell survival suppression, Immunotherapy

## Immune Suppression, Extracellular Vesicles, and Cancer

Tumor-induced immune suppression has been recognized as one of the major barriers for cancer immune therapies, including checkpoint inhibitors [1]. Immunosuppressive mechanisms that tumors utilize to silence anti-tumor immune cells are numerous and differ between tumor types. They may include overexpression in tumor tissues of inhibitory receptor/ligands (e.g., PD-1/PD-L1); production by tumors of various soluble factors such as IL-10, TGF-β, IL-35, IDO, arginase, galectin 9, adenosine, COX-2, PGE2, and/or oxygen radicals; accumulation in the TME of metabolic alterations such as, e.g., glucose deprivation; enhanced activation of immune regulatory cells, e.g., Treg, myeloid derived suppressor cells (MDSC); the MHC class I down-regulation/loss or inactivation of β2-microglobulin on tumor cells; and other mechanisms of the immune evasion as previously reviewed [2,3]. Among various molecular and/or genetic changes that human tumors inflict on immune cells, the production by tumor cells of extracellular vesicles (EVs) is emerging as a novel and still poorly understood mechanism of immune suppression.

All cells release EVs but cells under stress, including tumor cells growing in the hypoxic, acidic, and glucose-deprived environment or tumor cells treated with chemotherapy, produce more EVs than normal cells [4,5]. Endoplasmic reticulum (ER), the key organelle responsible for maintaining homeostasis, is stressed in tumor cells by intrinsic activation of oncogenes or extrinsic factors, including hypoxia, limited nutrient availability, acidosis. The ER stress leads to accumulations in tumor cells of misfolded proteins (the unfolded protein response or UPR) and to up-regulated expression of the stress proteins, IRE-1, ATF6, PERK [6]. Stress proteins initiate signaling of the major cellular pathways which either restore homeostasis or result in tumor cell death. The upregulation of stress proteins culminates in mitochondrial dysfunction and tumor cell-mediated intrinsic apoptosis [7]. Unless these proteins can be disposed of by either lysosomal processing or packaging into exosomes, tumor cell will die [7]. Thus, to survive and decrease the ER stress, tumor cells intensively produce and release exosomes packaged with stress proteins among other cellular components, many of which have immunoregulatory properties [3,8,9]. This explains why levels of plasma exosomes are substantially higher in patients with cancer than in healthy donors [4]. It has been suggested that the elevated numbers of EVs in plasma might serve as a biomarker of disease progression in cancer [4], a notion that requires further studies and confirmation.

EVs isolated from cancer patients’ plasma contain a subset of small EVs (sEV) derived from the endocytic compartment of parent tumor cells that are called “TEX.” These tumor-derived exosomes account for various proportions of the total exosome fraction in body fluids of cancer patients, depending on disease stage and activity. TEX carry unique genetic and molecular cargos of proteins, lipids, DNAs and RNAs that resemble parent tumor cells and thus can be distinguished from EVs derived from non-malignant cells. Since TEX, like other sEV, move freely in body fluids and tissues [10,11], they are viewed as a communication system between the tumor and non-malignant cells, including immune cells [12]. TEX, serving as miniature surrogates of tumor cells, carry active components of the immunosuppressive molecular pathways tumors operate (e.g., adenosine, TGF-β1, arginase-1, cytokine/chemokine networks and others, as reviewed in ref 2) that are known to be involved in promoting carcinogenesis [13,14]. TEX also transfer and deliver nucleic acids, DNA, long non-coding RNA, miRNAs and circulating RNA, which regulate gene expression and signaling pathways in recipient immune cells [12]. TEX derived from different cancer cell types carry distinct components, so that a given protein profile or miRNA profile may be unique for each vesicle depending on its origin and biogenesis in parent tumor cells. Importantly, TEX carry and deliver these diverse components to various types of recipient cells, inducing a re-organization of the cellular milieu and alterations in cellular functions [15]. These alterations are likely to be diverse in different recipient cells and involve transcriptomic, proteomic and/or epigenetic signals that culminate in reprogramming. However, the ability to alter functions of the recipient cells, including immune cells, to pro-tumor activities is the distinct characteristic of TEX that is not shared by sEV derived from non-malignant cells. This reflects the similarity of TEX molecular/genetic content with that of parent tumor cells and provides a platform for future use of TEX as biomarkers for cancer progression, staging or response to treatment [16]. As a potential component of liquid tumor biopsy, TEX offer special advantages as cancer biomarkers: they not only serve as noninvasive molecular/genetic mimics of tumor cells but also report on the degree of functional reprogramming induced in immune cells (or other cells) by the progressing tumor.

TEX carry and can deliver both suppressive and stimulatory signals to immune cells, and the outcome of their crosstalk with recipient cells may be “contextually” regulated. Thus, in cancer patients with advanced malignancies, TEX are enriched in the immunosuppressive cargo and are largely engaged in immunosuppressive pro-tumor reprogramming [17]. Mechanistically, the TEX cargo of lipids, proteins (i.e., enzymes, cytokines, various metabolites), and nucleic acids (i.e., DNA, RNA and microRNA) is transferred to recipient cells, where protein incorporation into cellular repertoire and gene transcription/translation, respectively, of delivered materials take place. The emerging data suggest that this transfer may results in: (i) metabolic reorganization; (ii) mitochondrial dysfunction and intrinsic apoptosis; (iii) activation of autophagy in an attempt of the recipient cell to rescue itself; and (iv) packaging of the harmful proteins into sEV and massive vesiculation of the recipient cell derived exosomes into extracellular space that is necessary for survival. The TEX uptake by immune cells and resulting molecular/genetic reprogramming may vary depending on the type of immune cells engaging in the crosstalk with TEX and may conceivably result in immune stimulation rather than suppression. TEX interact with all types of immune cells [18]. TEX interactions with and reprogramming of B cells, NK cells monocytes and dendritic cells (DCs) in cancer and other diseases have been extensively reviewed [19]. Here, the interaction of TEX with T cells is discussed focusing on the newly acquired mechanistic insights into TEX-mediated transfers of proteins or nucleic acids to T cells. The consequences of this transfer that may have therapeutic significance are emphasized.

## TEX Engagement and Entry into T cells

All EVs, including TEX, circulate freely and cross all tissue barriers, including the blood brain barrier (BBB) [10,11]. Numbers of circulating TEX depend on the rate of their production by tumor cells and their removal by the host reticuloendothelial system [20]. TEX numbers in human blood vary broadly among individuals. Estimates for circulating EVs in patients with cancer vary between 1×10^11^ to 1×10^12^/mL plasma [21], exceeding the EV numbers in healthy donor s(HDs) estimated at ~5×10^9^/mL plasma. These estimates suggest that EVs outnumber circulating white blood cells (WBCs) in the circulation, providing an opportunity for numerous EVs to engage a single WBC and establish crosstalk.

Much of what is known about TEX and their interactions with recipient cells comes from experiments performed with tumor cell lines, where all vesicles recovered from cellular supernatants are TEX. With cancer patients’ body fluids, TEX represent a variable subset of total circulating sEV from which they have to be separated for phenotypic or functional studies. Upon co-incubation with activated primary or cultured human T cells, TEX carrying surface associated adhesion molecules, integrins and a variety of immunoregulatory receptor/ligand proteins, encounter T cells which surface membranes are richly decorated by complementary recognition molecules. A recently published study explored the mechanisms of EVs-T cell interactions using TEX produced by triple negative breast cancer (TNBC) cell lines, MDA-MB-231 and MDA-MB-436, and human activated immune cells (mainly primary or cultured T cells) as an experimental model [9]. TEX were labeled with the PKH26 dye and their uptake by CD8^+^ and CD4^+^ T cells was monitored by fluorescence microscopy and flow cytometry. The initial TEX interaction with the surface membrane of recipient T cells was slow (10–15 min) relative to that previously measured in B cells, NK cells, or monocytes [22] and was followed by TEX uptake and their entry into the cytosol. This event may have involved one or several mechanisms such as receptor-ligand recognition, fusion or endocytosis, and by 1–2 h of co-incubation, nearly all T cells internalized numerous labeled TEX. The slow and prolonged uptake of TEX by a recipient T cell could potentially favor the receptor/ligand signaling that drove the TEX internalization. Interestingly, the TEX uptake by T cells was only partially inhibited (40–45%) by heating the vesicles to 80°C for 1 h or treating them with proteinase K, and was not blocked by pre-treatments with neutralizing anti-Fas Abs. Also, blocking TEX internalization with Dynasore or PitStop 2, which inhibit clathrin-mediated endocytosis, or with Cytochalasin D, an inhibitor of micropinocytosis, reduced by ~50% but did not eliminate TEX entry into T cells. These results suggested that mechanisms responsible for TEX entry into T cells are in part resistant to protein denaturation/digestion or to the blockade of endocytosis/micropinocytosis and might be largely mediated via receptor-ligand type of signaling. Importantly, the above-described attempts to block TEX entry only reduced but did not eliminate TEX induced apoptosis of recipient T cells, suggesting that once TEX engage receptors/ligands on the T cell surface, molecular signaling they deliver cannot be stopped and leads to apoptosis [9].

## Activated Human CD8+ T cells are Sensitive to TEX Induced Apoptosis

Numerous *ex vivo* studies of circulating lymphocyte subsets in patients with various types of cancer have suggested that the peripheral T lymphocyte depletion frequently observed in patients with advanced cancers [23] is induced by EV-mediated signaling via the death receptor/ligands, specifically Fas/FasL and TRAILR/TRAIL [24]. In these early studies, about 20% of T cells crosstalking with TEX showed DNA fragmentation [25]. More recent studies report the prominent presence of exhausted T cells in the circulation of patients with cancer [26]. These T cells produce little or no IL-2, IFN-γ or TNF-α and overexpress immune checkpoint proteins PD-1, LAG-3, TIM-3, TIGIT, CTLA-4 and others [26]. They are highly sensitive to apoptosis mediated by TEX which, like parental tumor cells, carry the respective reciprocal ligands: PD-L1, MHC class II, galectin-9, CD155 (poliovirus receptor) or B7 proteins, on their surface membranes [27]. These immunosuppressive ligands carried by TEX are not mutually exclusive in their ability to silence T cells. In fact, they may act simultaneously resulting in the profound inhibition of multiple effector functions in T cells that culminate in apoptosis. The TEX ability to initiate immunosuppressive checkpoint signaling in activated T cells was illustrated by co-incubation of TEX obtained from supernatants of MDA-MB-231 and MDA-MB-436 or with sEV isolated from plasma of patients (Pts) with TNBC [9]. The phenotypic surface profiles of TEX and sEV were determined by on-bead flow cytometry and their ability to induce apoptosis in Annexin V/PI binding assays. Phenotypically, TEX or sEV produced by the TNBC Pts had significantly higher levels of PD-L1, Fas, FasL, TRAIL and CTLA-4 as well as CD40, CD40L and OX40L than sEV produced by non-malignant HaCaT cells or sEV from plasma of healthy donors (HDs). Similarly, sEV isolated from plasma of TNBC Pts had significantly upregulated expression of the checkpoint proteins and elevated CD40L levels relative to HD’s sEV [9]. The scores calculated for suppressive proteins were significantly higher and those for stimulatory proteins significantly lower for TEX and for sEV from plasma of TNBC Pts. Apoptotic activity of TEX and sEV from plasma of TNBC Pts against activated primary human CD8^+^ T cells was significantly elevated relative to sEV from HaCaT or sEV from HD’s plasma. Interestingly, activated primary B and NK cells were relatively resistant to TEX-mediated apoptosis for reasons that are not clear but could relate to the signal strength or type that is necessary for inducing their apoptosis. For example, sEV in plasma of melanoma or AML patients were previously shown to suppress functions of NK cells by mechanisms that involved signaling via the NKG2D-MIC/AB rather than inhibition via the checkpoints T cells recognize on TEX [28]. In aggregate, these data suggested that TEX and plasma derived sEV of TNBC Pts were armed in complementary death receptor/ligands and checkpoint proteins which effectively induced apoptosis of activated CD8^+^ effector T cells. In contrast, vesicles from cultured non-malignant cells or sEV from HDs’ plasma carried fewer checkpoint proteins and mediated significantly lower apoptosis of effector T cells. Interestingly, while TEX co-incubation with primary activated CD8^+^ T cells or CD8^+^ Jurkat T cells for 6h induced concentration dependent apoptosis in ~50% of recipient CD8^+^ T cells, only ~30% of recipient CD4^+^ T cells were apoptotic. We have previously reported that TEX reduced STAT 5 phosphorylation in activated CD8^+^ T cells but upregulated STAT5 phosphorylation in CD4^+^ T cells, an indication of differences in signaling TEX may induce in different subsets of T cells [29].

**Figure 1.**
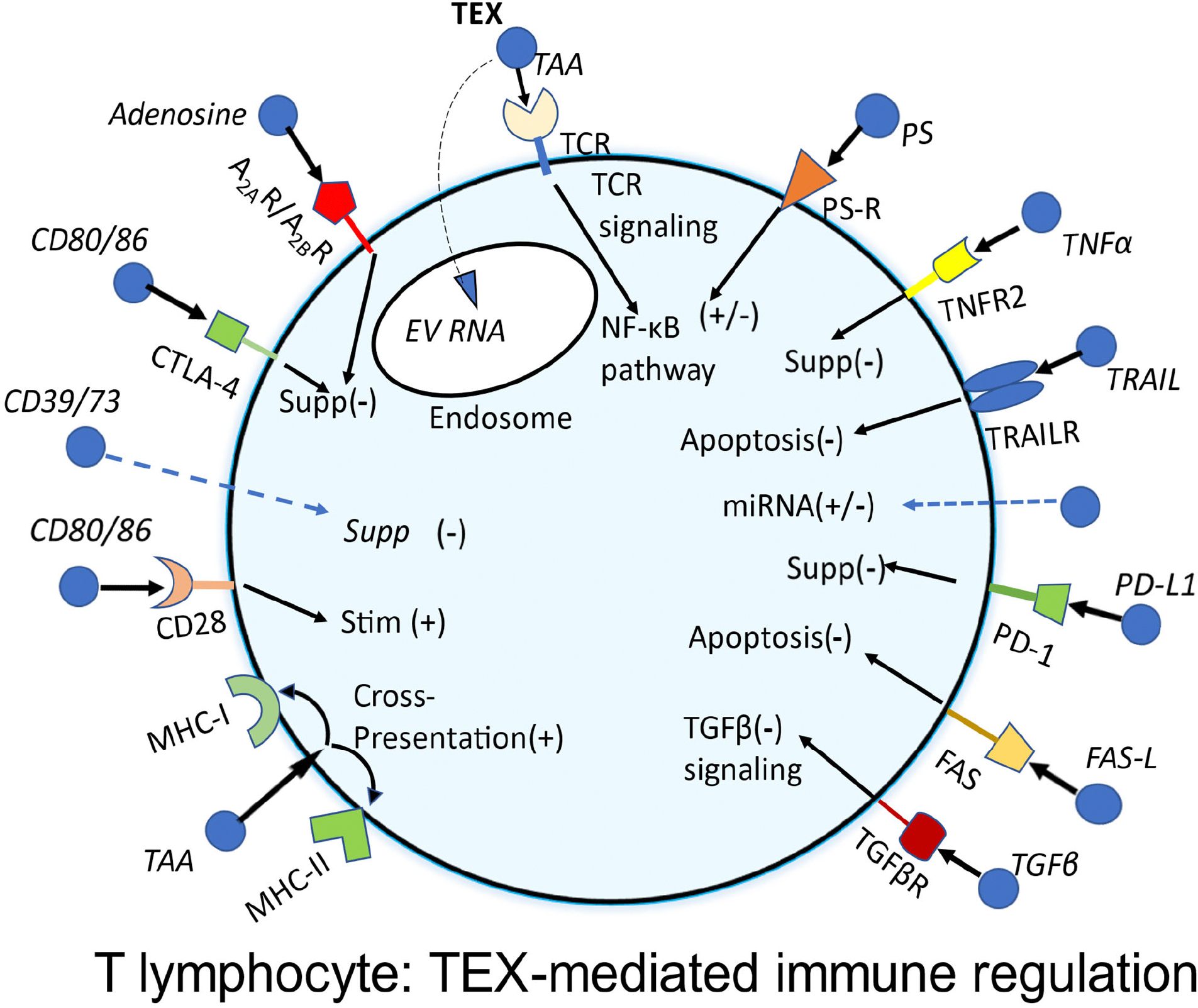
An image of a T cell interacting with various TEX (blue vesicles) carrying immunoregulatory proteins on the surface membrane. TEX bind to complementary receptors expressed by the T cell and initiate immunoregulatory signals which result in immune downregulation [Supp(−)] or immune stimulation [Stim(+)]. The sum of these simultaneously delivered signals will determine whether TEX mediate immune suppression or immune stimulation in the recipient T cell. Note that a single sEV might carry multiple signaling proteins on its surface membrane. Reproduced with minor changes from ref [9].

Differential expression of receptor/ligand proteins on the surface of immune cells and complementary receptors/ligands in TEX could in part explain the sensitivity/resistance of CD8^+^ T cells to TEX-mediated apoptosis. Therefore, expression levels of various signaling receptors (PD1, CTLA4, Fas, CD39, CD73, CD40, OX 40, TNFR, DR4, DR5) on the surface of activated T cells and of the complementary ligands (PD-L1, CD80, FasL, TRAIL, CD40L, OX40L) on the surface of TEX just prior to their co-incubation with T cells were determined by flow cytometry [9]. The heat maps of phenotypes for these TEX and sEV showed only moderate enrichment in all immunoregulatory proteins relative to HD’s sEV but not the expected increased expression of death receptor/ligand proteins that would explain high levels of apoptosis these sEV induce in recipient T cells. The image of a T cell responding to TEX/sEV signaling that emerged from this analysis suggests that the presence and expression levels of many different complementary receptors/ligands on surfaces of both partners regulate responses of recipient T cells to sEV signaling (Figure 1).

The model of TEX mediated immune regulation in Figure 1 shows a responder T cell receiving various simultaneously delivered signals from numerous (think millions of) sEV surrounding it in the circulation of a cancer patient. The sum of these simultaneously delivered regulatory signals determines whether suppression or activation of the T cell takes place. In the TME, where suppressive signals dominate, TEX-driven T cell reprogramming culminates in apoptosis.

## TEX Induced Apoptosis was Not Blocked by Neutralizing Death Receptor/Ligand Abs

Internalization of TEX by T cells takes ~10–15 min, as measured by flow cytometry [9,22]. This relatively slow rate of TEX uptake into activated T cells may be interpreted as evidence for the receptor/ligand type of entry that potentially could be inhibited by neutralizing Abs or pharmacologic inhibitors specific for the proteins carried by TEX e.g., PD-L1, FasL, CTLA-4, TRAIL or TGF-β. However, only minimal inhibition (~10% of isotype control) of TEX entry was observed with the neutralizing Abs or pharmacological inhibitors used alone or in combination, and T cell apoptosis was not inhibited [9]. Thus, neither blocking with neutralizing Abs nor digestion or denaturation of surface proteins involved in TEX-T cell interactions eliminated TEX-induced T cell death, although apoptosis was variably but not completely reduced in all cases. As already described above, interference with the TEX uptake by recipient T cells using pre-treatments with PK, heat, inhibitors of clathrin-mediated endocytosis (Dynasore or PitStop) or micropinocytosis (Cytochalasin D) only partially inhibited TEX entry and reduced T cell apoptosis to ~50% of controls but did not eliminate it. These data suggested that signals delivered by TEX binding to the surface of activated T cells drive persistent apoptosis in the recipient T cells and cannot be stopped or eliminated by interfering with activity of death receptor/ligands on the cell surface or with proteins mediating vesicle entry into the cytosol.

## TEX Induce Intrinsic Apoptosis in Recipient T cells

A possibility existed that the persistent TEX-induced apoptosis of T cells that could not be stopped by imposing various blockades was mediated by mechanisms introduced to recipient T cells by TEX. For example, it was possible that TEX delivered caspases, factors activating caspases or inhibitors of survival proteins to recipient T cells. However, the pre-treatment of recipient T cells with Pan-caspase or Caspase-8 inhibitors had no effect on the TEX entry and reduced apoptosis by ~30% but did not eliminate it. Expression levels of survival proteins, Bcl-2, BcL-xL and FLIP, were decreased in these recipient T cells, indicating that TEX altered mechanisms regulating survival of responder T cells. To further elucidate these mechanisms, T cells co-incubated with TEX for 3–16 h were permeabilized, lysed and separated into the mitochondrial and cytosol fractions [9]. Western blots of the mitochondrial fraction showed a progressive decrease in the levels of cytochrome C, Smac, Bcl-2 and Bcl-xL during the co-incubation with TEX. In the cytosol, levels of cytochrome C and Smac increased, and cleavage of caspase-3 (but not of caspase-8) and PARP were already evident at 3 h of incubation and increased progressively with the co-incubation time. Importantly, apoptosis inducing factor (AIF), which initiates caspase-independent intrinsic apoptosis by DNA fragmentation, was also released from mitochondria during co-incubation with TEX. The data showed that TEX taken up by activated T cells induced intrinsic apoptosis in recipient T cells characterized by AIF, cytochrome C, Smac, Bcl-2, BcL-xL release from mitochondria and the accumulation of these proteins in the T cell cytosol. sEV produced by non-malignant HaCaT cells co-incubated with T cells did not induce protein leakage from mitochondria.

In view of the evidence for TEX-induced intrinsic apoptosis in T cells, the failure to arrest this apoptosis by blocking death receptor/ligand signaling is not surprising. The data indicated that ~10% of apoptosis in responder T cells could be attributed to extrinsic apoptosis driven by signaling via death receptor/ligand proteins located on the T cell surface [9]. The question arises of how TEX signaling at the surface of a T cell leads to mitochondrial dysfunction and results in intrinsic apoptosis. One explanation might be that the physical stress created by vesicles entering recipient T cells rather than receptor/ligand-mediated signaling are responsible for the downstream apoptosis. Stress inducing stimuli are known to promote molecular alterations called stress associated molecular patterns or SAMPS [30]. Delivery of TEX which recapitulate the content of tumor cells, carry a broad variety of proteins, including stress proteins, and exhibit prolonged kinetics of entry into T cells are likely to induce SAMPS. Our recent RNAseq data indicate that T cells co-incubated with TEX for 4h upregulated PERK transcripts, and the proteomic analysis and Western blots of T cells co-incubated with TEX for 6 h confirmed upregulation of IRE1, PERK and ATF6 expression (Whiteside TL, unpublished). Since mitochondria are highly sensitive to cellular stress, it appears that TEX entering a T cell induce SAMPs, alter mitochondrial integrity and expression levels of survival proteins, leading to the unavoidable, relentless T cell death that cannot be stopped by interference with external receptor/ligand signaling [9].

## Implications of TEX-mediated Intrinsic Apoptosis of Activated T cells for Cancer Immunotherapy

The evidence that EV-mediated apoptosis of circulating CD8^+^ effector T cells in various cancers positively correlates with disease stage, activity, and cancer progression has been well documented [31]. This has led to a conclusion that silencing of TEX-driven apoptosis of effector T cells could benefit cancer patients, specifically those treated with checkpoint inhibitors. Thus, efforts to silence TEX and their immunosuppressive and pro-tumor activities have been of special interest to immuno-oncologists. Current approaches to immunotherapy are based on the notion that favorable therapeutic responses are associated with a robust recovery of endogenous T cells from tumor-induced immune suppression. However, the restorative effects of immunotherapy are likely to be counterbalanced by the presence in body fluids of cancer patients, especially those with advanced disease, of immunosuppressive TEX. Emerging evidence discussed above indicates that TEX-mediated crosstalk between cancer cells and immune cells contributes to dysfunction or exhaustion of effector CD8^+^ T cells, increasing their susceptibility to apoptosis [9,17]. Targeting with neutralizing Abs to eliminate negative signaling via the cognate death receptors/ligands on the surface of T cells interacting with TEX looked like a promising strategy that potentially could work in a therapeutic setting. The rationale was that restraining of negative TEX signaling prior to immune therapy would foster the recovery of immune cells and facilitate a repopulation with highly effective anti-tumor effector T cells. However, the data presented above eliminate signaling via extrinsic death receptor/ligands as a major mechanism of T cell demise by TEX-mediated apoptosis. Instead, the data introduce intrinsic apoptosis precipitated by unrestrained entry of TEX into the T cell cytosol, activation of stress pathways, mitochondrial dysfunction and reorganization of survival proteins as a major mechanism used by TEX for T cell re-programming and effector T cell elimination.

The ability of TEX to induce relentless intrinsic apoptosis, which is largely independent of death receptor/ligand signaling, in activated T cells represents a special danger to anti-cancer adoptive therapies. This danger is well illustrated by a small phase I clinical trial with NK-92 cells (Neukoplast; IND-BB 84040) offered some years ago to patients with refractory/relapsed AML [28]. The therapeutic failure of this trial was linked to the presence in very high numbers of highly immuno-suppressive TEX in the patients’ peripheral blood prior to adoptive immunotherapy. These TEX interacting with adoptively transferred NK-92 cells, rapidly downregulated expression of NKG2D on the NK-92 cell surface, significantly reduced NK-92 cell anti-leukemia activity and thus eliminated an opportunity for a therapeutic response to immunotherapy [28]. Adoptively transferred activated T cells or engineered CAR T cells might be especially vulnerable to TEX-mediated apoptosis. Preliminary data suggest that excessive numbers and destructive anti-immune cell activity of TEX in body fluids of patients with cancer might attenuate therapeutic benefits of checkpoint inhibitors and may partly contribute to the lack of response seen in some of the patients receiving immunotherapy. TEX emerge as a novel therapeutic target in cancer immunotherapy. Although the danger of TEX for activated T cells cannot be removed by blocking with neutralizing Ab, the possibility that TEX could be silenced by blocking their production is considered. However, strategies and technologies for a selective blockade of TEX production by tumor cells without the impairment of sEV production by non-malignant cells are not yet available. The enigma of “bad” TEX and “good,” homeostasis-regulating sEV circulating in the same cancer host may be difficult to address. Strategies for the amelioration of negative TEX activity to improve effects of immunotherapy await the development of drugs able to selectively inhibit TEX production by tumor cells. There is a some evidence that targeting calcium flux in tumor cells might work [32]. However, currently available methods for sEV isolation, separation of TEX from non-TEX in clinical samples and monitoring of sEV phenotype/activity at a single vesicle level are imperfect and create a barrier to research and development. Nevertheless, novel insights into TEX-mediated dysregulation of immune cell functions that are rapidly becoming available in *in vivo* tumor models provide us with a better understanding of the mechanisms of intercellular vesicular activity and offer hope for future translation of TEX silencing to the clinic.

In closing, it is important to remember that while TEX may be “bad actors” in the context of immunotherapy, they are of great interest as components of liquid tumor biopsy. Non-invasive biomarkers of cancer progression or response to therapy that reflect the changes in the tumor content and/or predict resistance to immunotherapy are an unmet need. TEX have a potential to meet this need, and profiling of TEX cargos by proteomics or transcriptomics offers a window of opportunity for validation of TEX as cancer biomarkers that potentially may exceed circulating tumor cells (CTC) and circulating free DNA (sfDNA) in the detection of minimal residual disease (MRD) or in predicting responses to therapy.
